# Decontamination of Spores on Model Stainless-Steel Surface by Using Foams Based on Alkyl Polyglucosides

**DOI:** 10.3390/molecules28030936

**Published:** 2023-01-17

**Authors:** Carolina Dari, Heni Dallagi, Christine Faille, Thomas Dubois, Christelle Lemy, Maureen Deleplace, Marwan Abdallah, Cosmin Gruescu, Julie Beaucé, Thierry Benezech, Anne-Laure Fameau

**Affiliations:** CNRS, INRAE, Centrale Lille, UMET, University Lille, 369 Rue Jules Guesde, F-59000 Lille, France

**Keywords:** foam, spore, cleaning, surface hygiene, life cycle analysis

## Abstract

In the food industry, the surfaces of processing equipment are considered to be major factors in the risk of food contamination. The cleaning process of solid surfaces is essential, but it requires a significant amount of water and chemicals. Herein, we report the use of foam flows based on alkyl polyglucosides (APGs) to remove spores of *Bacillus subtilis* on stainless-steel surfaces as the model-contaminated surface. Sodium dodecyl sulfate (SDS) was also studied as an anionic surfactant. Foams were characterized during flows by measuring the foam stability and the bubble size. The efficiency of spores’ removal was assessed by enumerations. We showed that foams based on APGs could remove efficiently the spores from the surfaces, but slightly less than foams based on SDS due to an effect of SDS itself on spores removal. The destabilization of the foams at the end of the process and the recovery of surfactant solutions were also evaluated by using filtration. Following a life cycle assessment (LCA) approach, we evaluated the impact of the foam flow on the global environmental footprint of the process. We showed significant environmental impact benefits with a reduction in water and energy consumption for foam cleaning. APGs are a good choice as surfactants as they decrease further the environmental impacts.

## 1. Introduction

Reducing food waste is one of the prominent goals set by the United Nations to achieve a more sustainable world by 2030. Indeed, food waste is responsible for unnecessary greenhouse emissions and waste of resources [1]. Therefore, limiting food waste is of major interest to limit climate change and ensure sustainability. An important contributor to food waste is microbial spoilage. Nearly one-third of all food produced worldwide is estimated to be lost mostly due to microbial spoilage after postharvest [1]. In the food industry, it is well known that surfaces in processing equipment can be contaminated by microorganisms despite cleaning and disinfection procedures [2]. Once present, a large portion of these bacteria persists on surfaces depending on the environmental conditions and often produces biofilms. Thus, food contact surfaces in processing equipment are considered a major risk factor for food cross-contamination. The formation of bacterial biofilms on food-processing equipment and food-contact surfaces can act as a persistent source of contamination of food, threatening the safety of food products, which can result in foodborne diseases, financial losses, and a loss of credibility for the company [3]. In addition, bacteria spores are of major concern to the food industry because a number of species are pathogenic, whereas others are associated with spoilage. Spores also have the capacity to adhere to materials of importance in the processing industries, such as stainless steel widely used in food equipment manufacturing [4]. The cleaning phase is the most important stage for minimizing microbial colonization and removing attached microorganisms. For example, the efficiency of cleaning and sanitation of milk contact surfaces are widely influenced by many factors such as the nature of contamination, the microtopography of surfaces, the design of the equipment, and the cleaning conditions [5]. Cleaning-in-place (CIP) is the ubiquitous process used to maintain the hygienic state of the processing lines by cleaning their interior surfaces (pipelines and equipment) without dismantling the facilities. In the CIP process, cleaning solutions used include various types of detergents. CIP usually involves the circulation of cleaning solutions at high temperatures through the plant under conditions of increased turbulence and flow speed. Therefore, the effectiveness of cleaning is preconditioned by four main factors: chemical agent, mechanical power, temperature, and time of the procedure, which together form the Sinner circle [6]. However, the environmental impact of the CIP process is significant, as frequent cleaning is required and water is used intensively along with chemicals. For example, in the dairy industry, most of the water consumption is directly linked to a CIP between 0.6 and 6 L of water per liter of treated milk [2]. In addition, CIP generates large quantities of wastewater, with an additional economic burden on the industry, and an environmental burden on the community [7]. Hence, the food industry is currently looking for new cleaning processes to decrease the environmental impact.

Almost twenty years ago, foam circulation through pipes was described in the literature to be very efficient for radioactive surface decontamination of solid surfaces, because the amounts of chemicals involved and the volume of radioactive waste produced were much lower than with other processes [8]. Thus, one strategy described recently in the literature is the use of foam flows for cleaning in the food industry [9,10,11]. Due to foam wall friction and specific foam rheology properties in terms of viscous stress, foam bubbles could remove efficiently spores and biofilms [12,13,14]. In addition, foams enable the drastic reduction in the amount of water used because foams are mainly composed of air bubbles [15]. Therefore, the use of foams could have a direct positive impact on the environmental footprint of the cleaning process [9,10,11]. These first studies were based on the use of sodium dodecyl sulfate (SDS) as a surfactant at a concentration well below the CMC, leading to unstable foam [9,10,11]. The aim of this study was to show that a decrease in the environmental footprint of foam flow cleaning was possible through the use of alkyl polyglucosides surfactants (APGs). Indeed, APGs are biosurfactants produced from vegetable oils and starch (plant-derived feedstock chemicals) suitable for cleaning applications in food industries, but also already widely used for various applications in personal care products. Moreover, APG foams have been shown to be very efficient for spores decontamination under static conditions on vertical surfaces [16]. We studied two different APGs known to be good cleansing and foaming agents: decyl glucoside (DG) and lauryl glucoside (LG) [17,18]. Foams were characterized in terms of foamability, liquid fraction, and bubbles size. The efficiency of spores’ removal by foam flows was studied on stainless-steel surfaces and assessed by enumeration. The impact of the foam flow process on the global environmental footprint was determined by the life cycle assessment (LCA) approach, and we compared foams based on APGs with foam based on SDS. We also evaluated the destabilization of the foams by ultrasound and the removal of spores and the recovery of clean surfactant solution in order to regenerate the foam at the end of the process and to limit the waste water.

## 2. Results and Discussion

### 2.1. Model Surfaces, Spores, and Surfactant Solutions Characterization

In this study, we used stainless-steel plates as model surfaces and spores from *Bacillus subtilis* as the model contaminant to study the cleaning process by the foam flow. To mimic the surfaces encountered in the dairy industry, we followed a protocol described and shown in Section 3.1.2 to prepare the surfaces with different steps of soiling and cleaning [9]. The water contact angle was 45.1 ± 1.5, showing that the surfaces were hydrophilic. We also determined the hydrophobic/hydrophilic characteristic of the spores used by the MATH procedure. The aqueous affinity for the spores used in this study was higher than 90%, so they were considered as highly hydrophilic as it was also shown before [19]. To produce the foam, we used three different surfactants. Two alkyl polyglucosides: decyl glucoside (DG) and lauryl glucoside (LG), were used to study the effect of the alkyl chain length on the foam flow cleaning efficiency. The SDS was used as a reference as it was the surfactant used for the first study on spores’ decontamination by the foam flow [9]. The CMC of the three surfactants was determined in order to produce foam at a concentration close to the CMC. The CMC was estimated at 0.03 wt.% for DG, 0.03 wt.% for LG, and 0.23 wt.% SDS. These values are in accordance with the literature [17,20].

### 2.2. Effect of Surfactants on Spore Detachment under Static Conditions

**Figure 1:**
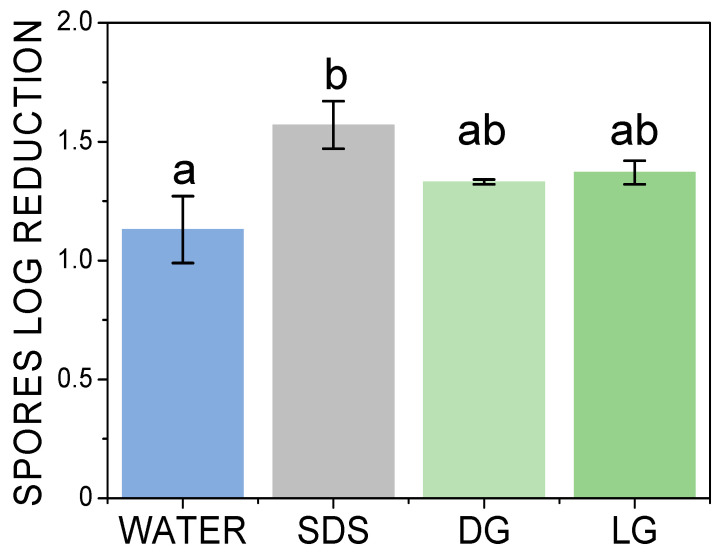
Spores log reduction on the plate under static dipping conditions as a function of the surfactant in comparison to water. The small letters a–b indicate groups of statistical differences according to Tukey’s test (*p* < 0.05).

Before studying the effect of foam flow cleaning, it was necessary first to study the effect of each surfactant solution on spore detachment under static conditions, that is to say, the chemical action. In this aim, the stainless-steel plates contaminated by the spores were directly dipped in the surfactant solution or in osmosis water for 30 min. Then, the remaining spores on the plates were enumerated (Figure 1). The spore log reduction was around 1.13 ± 0.14 for water, showing that dipping the contaminated plates in water results in more than one log removal of spores. This result suggested that some of the hydrophilic spores deposited on the surface of the plates were not strongly adherent [4]. For the two APGs, the spores log reduction was similar with a spores log reduction of 1.33 ± 0.01 and 1.37 ± 0.05 for DG and LG, respectively. These values were slightly higher than those for water alone, showing a slight effect on APGs on the spores’ removal under static conditions. For SDS, the highest value was obtained, with a spores log reduction of 1.57 ± 0.10. Thus, SDS had the strongest effect on spores’ removal under static conditions. We suppose that a chemical action of SDS is possible due to its anionic nature in comparison to APGs, which are non-ionic surfactants. In the literature, SDS is known to denature proteins even at room temperature and at sub-CMC concentrations [21]. Therefore, it is possible that in our experimental conditions, SDS could denature the spore surface proteins, thus facilitating the spores’ detachment from surfaces.

### 2.3. Effect of Foams on the Detachment of Spores

**Figure 2:**
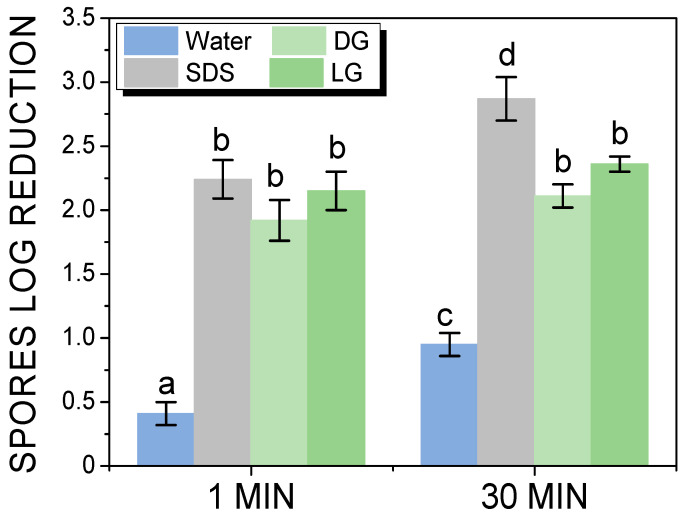
Spores log reduction on the plate in dynamic condition during foam flow cleaning after 1 and 30 min of foam flow as a function of the surfactant in comparison to water. The small letters a–d indicate groups of statistical differences according to Tukey’s test (*p* < 0.05) between the water and the surfactants for 1 min of foam flow cleaning and for 30 min of foam flow cleaning.

The effect of foam flow cleaning on spores’ removal from the stainless-steel plate was investigated by introducing the plate in a duct. Then, the effect of the foam flow was quantified by measuring the spore log reduction after one minute and 30 min of foam flow (Figure 2). After one minute of foam flow, the spore log reduction was around 0.41 ± 0.09 for the water, due to the hydrophilicity of the spores. The spore log reduction was 1.92 ± 0.16, 2.15 ± 0.15, and 2.24 ± 0.15, respectively, for DG, LG, and SDS after 1 min of foam flow. These values were much higher than for water alone, showing that foam flows efficiently removed spores from the plate. The foam flow resulted in a very quick detachment of the adhering spores, due to a mechanical effect. In addition, there was no effect of the surfactant used. After 30 min of water flow, the spores log reduction increased to 0.95 ± 0.09, confirming the effect of water on the spores’ removal as already shown under static conditions after 30 min of dipping in water. The two APGs had similar values of 2.11 ± 0.09 and 2.36 ± 0.06, for DG and LG, respectively, after 30 min. These values show that for APGs, the foam flow cleaning was efficient in the first minute, and it could not be improved by increasing the time of cleaning. For SDS, the spore log reduction slightly increased with the increase in time and reached 2.87 ± 0.17 after 30 min of foam flow. Thus, after 30 min of cleaning, the foam based on SDS was the most efficient to spores removal. Based on the effect shown of SDS on spore removal under static conditions, we suppose that the effect of the SDS foam flow could be a combination of foam flow and/or SDS chemical action on spore surface proteins. It is important to point out also that in our previous studies, the foams based on SDS always had a stronger effect on the spores’ removal than the surfactant solution whatever the foam properties in terms of bubbles size and foam flow velocity [9,10,11].

**Figure 3:**
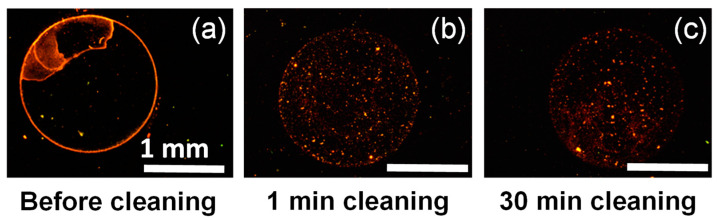
Epifluorescence microscopy pictures of a stainless-steel plate contaminated by spores and stained with acridine orange: (**a**) before cleaning by foam flow, (**b**) after one minute of foam flow cleaning based on DG, and (**c**) after 30 min of foam flow cleaning based on DG. The scale bar represents 1 mm for all the pictures.

The effect of foam flow cleaning on spore removal was also followed by epifluorescence microscopy before and after foam flow cleaning using the three surfactants. For all the foams, an efficient removal of spores was observed just after one minute of flow (Figure 3). No differences could be observed between the three surfactant foams, confirming the results of spore log reduction quantification. Moreover, in all cases, there was no visible difference between the pictures after 1 or 30 min of foam flow.

### 2.4. Foam Characterizations before and after Flowing Inside the Duct

**Figure 4:**
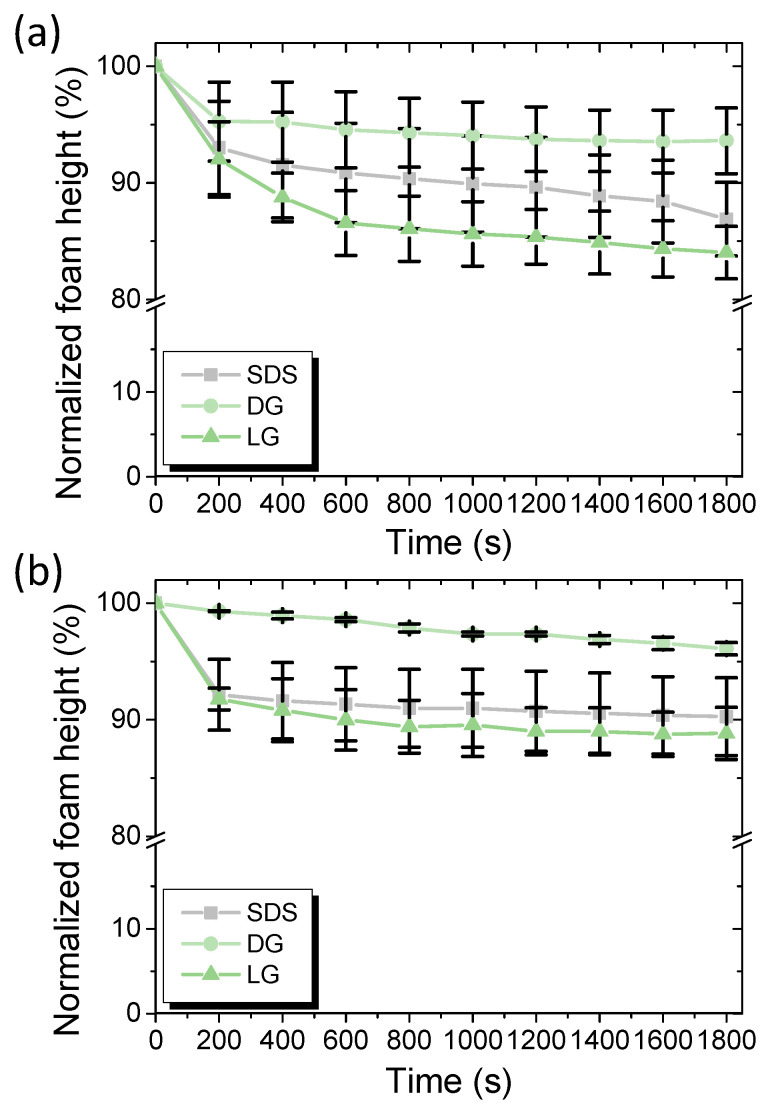
Evolution of the foam height for SDS, DG, and LG: (**a**) before the foam passed through the duct containing the plate contaminated with spores and (**b**) after the foam passed through the duct.

The characterization of foams during the flow inside the duct was very difficult to achieve in situ. That is why the foams based on SDS, DG, and LG were characterized in terms of foam stability under vertical conditions by measuring the drainage, the foam height, the mean bubbles area, and the bubbles size before and after the foam passed through the cleaning duct. Thus, we compared the foam before and after passing through the duct to obtain information on what happened to the foam during the flow in terms of foam stability (change in bubbles size and liquid fraction). It was important to determine if the foam properties changed during the flow and if it could explain the differences between the surfactants. The foams were sampled and immediately analyzed in vertical conditions using a glass column. The evolution of the normalized foam height was followed as a function of time to obtain insight into the foam stability (Figure 4). For the three surfactants before the foam flowed through the duct, a fast decrease in the foam height until 200–400 s was observed with around a decrease of 10% of the initial foam height (Figure 4a). Then, the foam height was stabilized around 1800 s and was 86.9% ± 3.1, 93.6% ± 2.8, and 84.0% ± 2.2 for SDS, DG, and LG, respectively. After the foam flowed through the duct, SDS and LG showed the same evolution for the normalized foam height (Figure 4b). However, the foam based on DG was slightly more stable. The foam height was stabilized around 1800 s and was 90.3% ± 3.3, 96.1% ± 0.5, and 88.8% ± 2.2 for SDS, DG, and LG, respectively. Before and after the duct, a relatively similar evolution was observed for the normalized foam height for the three surfactants. Before and after the duct, a very fast drainage was also observed until 200–300 s, for all the surfactants. The liquid fraction was measured directly for the three surfactants by sampling the foam before and after the duct, and in all cases, the three foams were considered wet foams with a liquid fraction above 5%.

**Figure 5:**
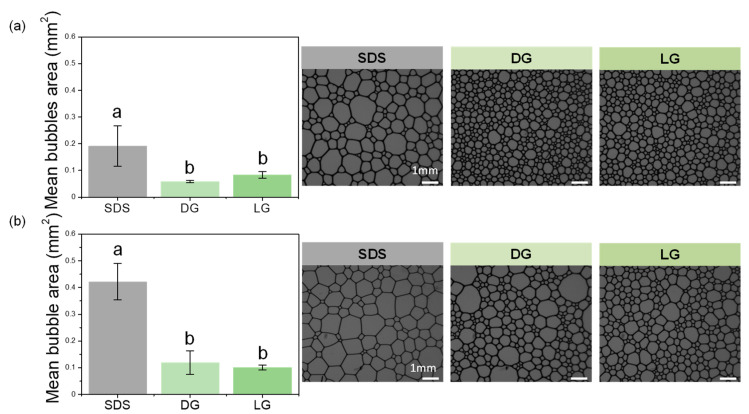
Mean bubbles area of the foams produced with SDS, DG, and LG with the corresponding microscopic pictures: (**a**) before the foam passed through the duct containing the plate contaminated with spores and (**b**) after the foam passed through the duct. The scale bar represents 1 mm for all the foam pictures. The small letters a–b indicate groups of statistical differences according to Tukey’s test (*p* < 0.05).

We compared the mean bubble area after 600 s for the three surfactants when the decrease in the foam height was almost stopped (Figure 5a,b). Before the duct, the mean bubble area was 0.19 mm^2^ ± 0.08, 0.06 mm^2^ ± 0.01, and 0.08 mm^2^ ± 0.01 for SDS, DG, and LG, respectively (Figure 5a). As shown also in the bubble pictures in Figure 5a, the bubble size was bigger for SDS than for DG and LG, which both showed similar bubble sizes. After the duct, the mean bubble area was 0.42 mm^2^ ± 0.07, 0.12 mm^2^ ± 0.04, and 0.10 mm^2^ ± 0.01 for SDS, DG, and LG, respectively (Figure 5b). The bubbles size was bigger for SDS than for DG and LG, which both showed similar bubble sizes (Figure 5b). The statistical analysis showed that there was a difference in the mean bubble area before and after the foams passed through the duct for the SDS but not for the APGs. Foams based on APGs were much more stable during the flow than foams with SDS. Based on previous results described in the literature on the foam wall friction, for the three surfactants, the foams were similar in terms of foam-wall friction as they were wet foams with quite similar air volume fraction. Moreover, the three surfactants used are known to produce mobile surfaces at the air/water surface [12,13,14]. Therefore, our results show that the efficiency of the SDS foams in spore removal from the flow in comparison to APGs foams came from the chemical action of SDS as demonstrated under static conditions (Section 2.2).

### 2.5. Foam Destruction and Recycling of Surfactant Solutions by Filtration

**Figure 6:**
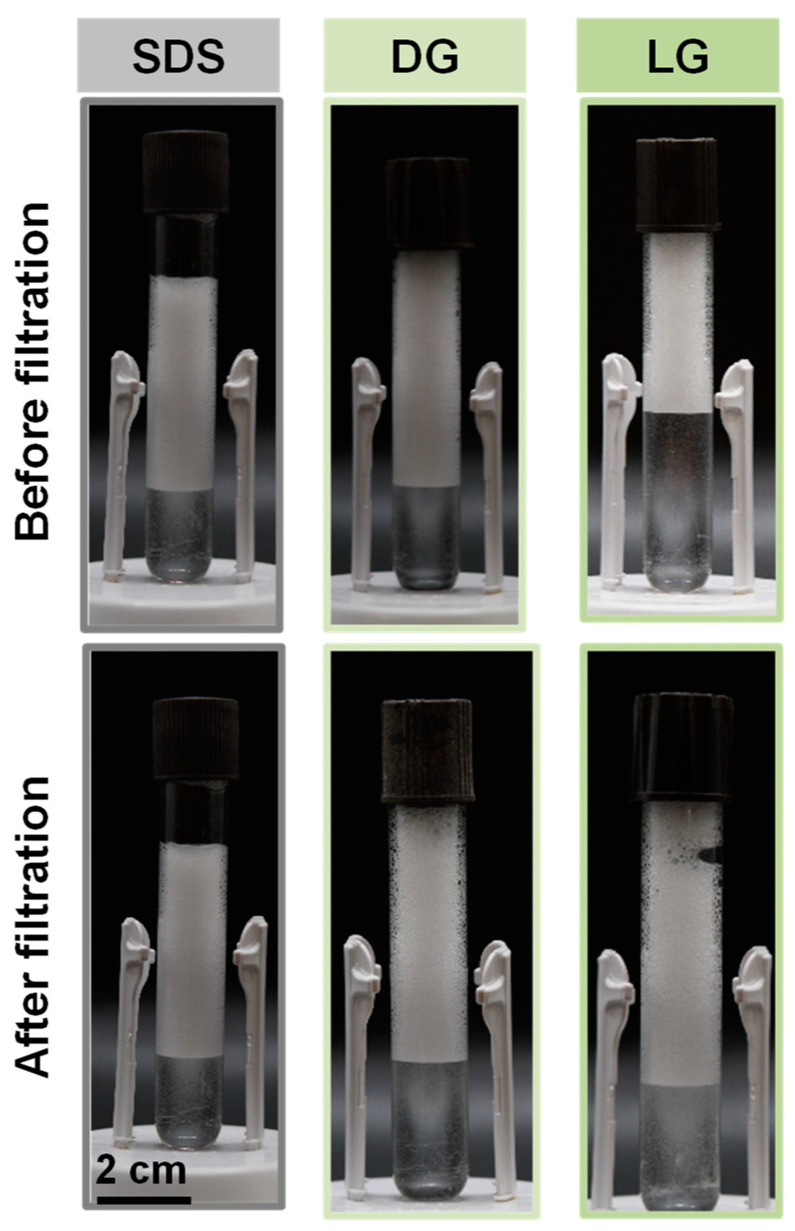
Pictures of the foams produced from the surfactants solution (SDS, DG, and LG) before and after filtration to remove the spores, showing a similar foamability and foam stability around 5 min after producing the foam. The scale bar represents 2 cm for all the pictures.

As already described for foams circulating through pipes for radioactive decontamination, the foam destruction at the end of the cleaning process and its regeneration is crucial [8]. Indeed, the aim of our foam cleaning process for the food industry is to generate foam, which then circulates through the ducts until the level of suitable cleaning is achieved. In order to efficiently destroy the foam on demand at the end of the process, the ultrasound technique seemed to be well adapted [22]. The ultrasound could be used at the end of the ducts as already described in the nuclear decontamination [8]. To regenerate the foam based on the same surfactant solution, perfect control of the decontamination of spores present inside the water was required. For this step, a filtration technique was used to illustrate how the spores could be removed. Filtration is widely used in the food industry and for the treatment of effluents and wastewater [23]. It has competitive advantages over other separation processes because it is easy to implement, flexible, compact, and automatic [24]. To determine the efficiency of the filtration step to decontaminate the spores from the surfactant solution, we quantified the number of spores present before and after the filtration. The results showed that for all surfactants solutions, the spore removal by filtration was very efficient as the number of spores after filtration was zero (below detection limit). Then, we compared the foaming properties of the surfactant solution before and after filtration by producing again the foam (Figure 6). As shown in Figure 6, the foam volume, stability, and appearance were the same for the three surfactants before and after filtration, showing that the filtration step was a good method to recycle the foaming surfactant solution without altering surfactant concentration and foaming ability. Thus, it would be possible after recovering the foam to destroy it by ultrasound, and the spores present inside the surfactants solution could be efficiently removed by filtration. The foam could then be regenerated based on the same surfactant solution during many filtration cycles (at least 10 cycles). In principle, the surfactant solution could be recyclable many times until the concentration of the surfactant solution would be too low due to the loss of surfactant during the cleaning and filtration steps.

### 2.6. Life Cycle Assessment of the Foam Flow Process: Comparison between SDS and APGs

**Figure 7:**
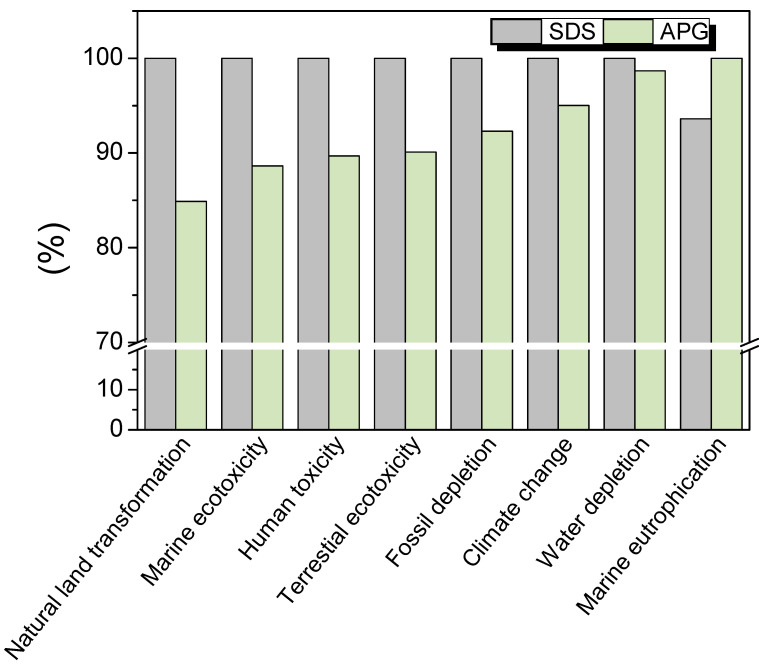
Environmental comparison between foam cleaning process with APG and foam cleaning process with SDS (reference system) by impact category (ReCiPe midpoint H).

The results of the LCA modeling obtained by the ReCiPe method are shown in Figure 7 and in Table S1 (in Supplementary File). We compared the foams studied previously with SDS (reference system) with foams produced with APGs [9,11]. It is important to notice that for the LCA analysis, we chose the fatty alcohol sulfate from renewable resources and not from petrochemical sources to reduce the possible impact of SDS [25]. In this study, for foams produced by both surfactants (SDS and APG), the contribution of the process parameters (water consumption for surfactant solution preparation, pump energy consumption, and compressed air consumption) contributed mainly to the impact of the process in the same way. The pump energy consumption seemed to be the major contributor to most of the impacts studied. By comparing the processes of cleaning with foam formulated with SDS and with APG, we observed that the most important impact of using APG instead of SDS was for natural land transformation (15% of reduction). For terrestrial ecotoxicity, the use of APG reduced the impact by around 9%. The negative impact of SDS for these two parameters came from the fatty alcohol sulfate used to produce SDS. This result was not surprising, as the fatty alcohol sulfate was reported to be a harmful contributor to most environmental impact indicators in previous LCA studies comparing different surfactants formulations for cleaning [25]. For marine ecotoxicity, human toxicity, fossil depletion, and climate change, the use of APG reduced them by around 11, 10, 8, and 5%, respectively. For these parameters, the fatty alcohol sulfate was the second most important contributor, after the pump energy consumption (present in both surfactant foam processes). For water depletion, the impact of using APG instead of SDS was very low, around 2%. For this parameter, the fatty alcohol sulfate was the third most important factor, after the pump energy consumption and the water consumption, which were present in both foam processes. For marine eutrophication, the use of APG increased the impact in a negative way in comparison to SDS (around 6% of increase). Although the main contributor to marine eutrophication was the energy consumption of the pump, which was found in both processes, the potato starch used to produce the APG had a negative impact. We can conclude that the most harmful process was the one formulated with SDS, as it had a major contribution to all impacts except marine eutrophication. From the LCA study, the foams based on APGs helped to further decrease the environmental impacts of the cleaning process in comparison to the foams based on SDS used in the previous studies, even when SDS synthesis is not entirely coming from petrochemical resources [9,11].

## 3. Materials and Methods

### 3.1. Materials

#### 3.1.1. Preparation of Surfactant Solution

Lauryl glucoside (Plantacare 1200) with 51.3 wt.% of active matter and decyl glucoside (Plantacare 2000) with 53.0 wt.% of active matter were provided by Ludwigshafen, BASF, Germany. Lauryl glucoside and decyl glucoside were used at a concentration of 0.1 wt.% of the active matter. Sodium Dodecyl Sulfate with 98.5% purity was purchased from Sigma Aldrich, Saint Quentin Fallavier, France. SDS was used at a concentration of 0.23 wt.%.

#### 3.1.2. Preparation of Solid Model Surfaces

**Figure 8:**
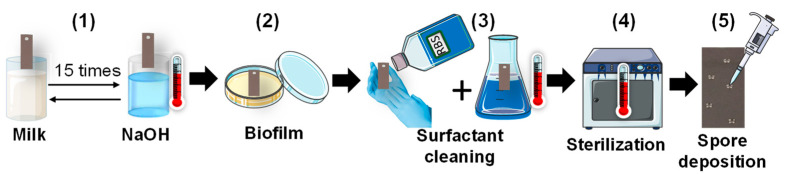
Different steps of stainless-steel plate surface preparation methodology: (**1**) 15 cycles of immersion of the plate in milk at room temperature for 30 min and in sodium hydroxide at 70 °C for 30 min; (**2**) biofilm formation on the plate; (**3**) rubbing of the plate with commercial undiluted surfactant (RBS) followed by immersion of the plate in surfactant aqueous solution at 5 wt.% at 60 °C for 10 min; (**4**) the plate sterilization in a hot air oven at 180 °C for 1 h; (**5**) soiling of the plate with 5 drops of 1 μL of the spore suspension at a concentration of 10^8^ CFU.mL^−1^.

All the cleaning experiments were carried out on rectangular (45 mm × 15 mm) AISI 316 stainless-steel plates (APERAM, Isbergues, France) with a 2R factory finish. In order to have similar surface properties to those found in the food industry, the surfaces were subjected to a conditioning process shown in Figure 8 [9]. The plates were immersed in milk at room temperature for 30 min and then they were rinsed under osmosis water for 5 min by overflow. The plates were immersed in a 0.5 wt.% sodium hydroxide solution at 70 °C for 30 min and then they were rinsed in osmosis water for 5 min. This cycle was repeated 15 times. Then, they were fouled with *Escherichia coli* strains previously shown to produce biofilms on stainless-steel surfaces [11]. For the biofilm formation, the plates were placed in a petri dish, and covered with 50 mL of a bacterial suspension consisting of 1/10 Tryptone Soy Broth (TSB, Biokar Diagnostics, Allonne, France) inoculated with 24 h of culture of *Escherichia coli* (final concentration around 10^7^ CFU.mL^−1^), and they were incubated at 30 °C for 24 h. Afterward, the plates were submitted to cleaning and disinfection steps. They were cleaned by rubbing the surfaces with an alkaline detergent RBS T105 (Chemical products, Brussels, Belgium) used usually for cleaning in the food industry [9]. They were immersed in a 5 wt.% RBS T105 solution at 60 °C for 10 min, followed by a rinse under running tap water for 5 min and then under osmosis water for 5 min. A period of 24 h before each experiment, the surfaces were sterilized in a dry heat oven at 180 °C for 1 h.

#### 3.1.3. Preparation of Spore’s Suspension

*Bacillus subtilis* PY79 is a laboratory strain known to produce hydrophilic spores. The *Bacillus subtilis* PY79 strain was tagged with green fluorescent proteins to make the spores fluorescent [19]. Frozen Bs PY79 was plated on nutrient broth agar (nutrient broth 13 g.L^−1^; agar 15 g.L^−1^) with an antibiotic (5 g.mL^−1^ of chloramphenicol) to promote the production of the green fluorescent protein, and incubated at 30 °C for 24 h. After 24 h, another plating was performed in the same conditions. The following day, some colonies were picked with a loop and inoculated in 50 mL of nutrient broth (Biokar Diagnostics, Beauvais, France) at 30 °C with continuous agitation for about 5 h until the absorbance at 600 nm was 1.2. Then, the suspension was centrifugated at 20 °C for 15 min at 1500× *g*. The amount of supernatant necessary was drained in order to leave a volume slightly greater than 200 μL per Petri dish to be seeded. The cell pellet was resuspended by shaking with a vortex and immediately used to inoculate the sporulation medium. The suspension was spread on Spo8-agar [26] and incubated at 30 °C for 10 days, when sporulation reached at least 95%. The spores were harvested by scraping the surface from the plates using sterile milli-Q water and washed five times in sterile milli-Q water by centrifugation (4 °C for 15 min at 3500× *g*) and resuspension.

To determine the spores’ hydrophilic character, we used the microbial adhesion to hydrocarbons method (MATH), which is based on the affinity of the spores for hexadecane [26]. The hydrophilic characteristic corresponds to a high percentage of affinity for the aqueous phase, while a high percentage of affinity for the hexadecane corresponds to a hydrophobic characteristic. The spore suspension (10^8^–10^9^ spores.mL^−1^) was re-suspended in 8.5 g.L^−1^ of NaCl aqueous solution to obtain an absorbance value of 0.5 to 0.6 at 600 nm (A_0_). Three milliliters of the spore suspension were put in glass tubes with 500 μL of hexadecane each and the tubes were vortexed at 2400 rpm for times ranging from 5 to 900 s. Then, each tube was left to settle for 30 min and then the absorbance of the aqueous phase of each tube was measured at 600 nm (A_t_). The percentage of affinity for the aqueous phase was calculated as follows (A_t_/A_0_) × 100 for each vortex time [26].

#### 3.1.4. Soiling of Surfaces with the Spores Suspension

The concentration of the spore suspension to soil the plates was set at 10^8^ CFU.mL^−1^. In order to avoid the presence of spore aggregates, the spore suspension was sonicated in an ultrasonic bath (Bransonic 2510E-MT, Branson Ultrasonics Corporation, Danbury, CT, USA) before each experiment for 2 min and 30 s. A total of 5 drops of 1 μL of the spore suspension were placed in each plate. Then, the plates were dried for 1 h at 30 °C.

### 3.2. Methods

#### 3.2.1. Determination of the Critical Micelle Concentration by Surface Tension Measurements

The critical micelle concentration (CMC) of APGs and SDS was estimated by surface tension measurements performed with a DSA100S drop shape analyzer (KRUSS, Hamburg, Germany) using the pendant drop method. The measurements were carried out at room temperature (23 °C ± 1.3 °C) with a stainless-steel needle 1 mm in diameter.

#### 3.2.2. Contact Angle Measurements of the Model Surface

The surface wettability was determined by measuring the contact angle of a water droplet (1 µL) deposited directly into the surface with the DSA100S Drop shape analyzer (KRÜSS, Hamburg, Germany). The temperature during the analysis was 20 ± 2 °C. All measurements were performed in triplicate and for two different plates.

#### 3.2.3. Spores Detachment Analysis

For the quantification of the adhered spores, each plate was sampled with a dry cotton swab (Copan, Brescia, Italy), which was then put in a tube containing 5 mL of sterile Milli-Q water. Each tube was vortexed for 1 min at 2400 rpm. Then, the swabs were removed from the tubes. Serial dilutions of the suspensions in the tubes were performed in sterile Milli-Q water and 100 μL of each dilution were plated in Tryptic Soy Agar (TSA; Biokar Diagnostics, Allonne, France). The plates were incubated for 24 h at 30 °C. The number of bacterial colonies was counted, and the results were expressed as the mean of the colony forming units plate^−1^ (CFU.plate^−1^). The cleaning efficiency was calculated by dividing the number of viable spores after the cleaning tests (whether static conditions or foam cleaning) by the number of spores on plates used as a control for the initial spore concentration. The cleaning efficiency was expressed in terms of log reduction using Equation (1):

*Log reduction = log (CFU.plate*^−1^*_t_−CFU.plate*^−1^_*t*0_) (1)

where *CFU.plate^−^*^1^ *_t_*: the mean number of the colony-forming units per plate after cleaning (*t* = 1 min or 30 min); *CFU.plate^−^*^1^*_t_*_0_: the mean initial number of the colony-forming units per plate. 

#### 3.2.4. Spores Detachment under Static Conditions

Three soiled plates were dipped vertically in 1 L of each surfactant solution or only in osmosis water for 30 min at room temperature. Three soiled plates were used as a control for the enumeration of the initial spore concentration. The experiments were carried out in triplicate.

#### 3.2.5. Production of Foams

The foam production device used in this study was the same as those used in previous works [9,10]. In brief, first, the surfactants were dissolved in osmotic water in the inlet tank of the foam production device. Before they were used for foam production, they were recirculated using a pump for five minutes, to ensure proper mixing. The surfactant solution was pumped into a tank, which was located at a 3 m height to ensure a constant flow due to gravity. The surfactant solution flowed at a flow rate of 4.5 L.h^−1^ into the foam generator, where the foam was produced by bubbling compressed air at a flow rate of 4.5 L.h^−1^ into the surfactant solution through a porous disc (pore size: 1–1.6 μm, DURAN, France). Then, the foam flowed through a horizontally placed square duct (1.5 cm high × 1.1 cm width × 23 cm in length) where the cleaning process was carried out.

The mean velocity of the foam (*v*) was around 1.5 cm.s^−1^ and it was calculated according to Equation (2):

(2) v=(Qs+Qa)/S

where Qs: the surfactant solution flow rate in L.h^−1^; Qa: the air flow rate in L.h^−1^; S: the duct cross-section in cm.

The foam production parameters were chosen based on our previous studies on SDS foams in order to have an efficient spore’s removal effect by producing foams with an adequate bubbles size and foam flow velocity [9,10,11].

#### 3.2.6. Effect of Foams on the Detachment of Spores

The foam flow cleaning efficiency was evaluated after 1 and 30 min of foam flow. For each time, four soiled plates (one for microscopy and three for the spore quantification) were introduced in a rectangular stainless-steel duct, which was then connected to the foam flow cleaning setup. Three extra soiled plates were used as a control of the initial spore concentration.

#### 3.2.7. Microscopic Observation of the Detachment of Spores

The microstructure of the spore drops placed on the plate surfaces was observed before and after cleaning. The spores deposited on the plates were stained with orange acridine at 0.01 wt.% and left for 15 min under dark conditions. Then, they were softly rinsed with osmosis water. Once they were dried, they were observed using an epifluorescence microscope at 50× magnification (Zeiss Axioskop 2 Plus, Oberkochen, Germany).

#### 3.2.8. Foams Characterization

The foams produced in the foam flow cleaning setup were characterized in terms of bubble size, foam height, and drainage. For the liquid fraction, we took a known volume of foam after the foam passed through the cleaning duct. Then, we measured the volume of liquid remaining when the foam was destroyed after a few hours. From the volume of liquid and the liquid of foam, we calculated the liquid fraction corresponding to the ratio between the two values. The height of the foams and the bubble size over time were measured using a dynamic foam analyzer-DFA100 (KRUSS, Hamburg, Germany). The foams samples were collected directly in the glass column (40 mm inner diameter) with a prism incorporated in the whole length of the column. For each foam, a sample was taken before and after the foam passed through the cleaning duct. The foams’ height and the bubbles’ size were simultaneously measured. The foams’ height was measured by using an LED panel and a line sensor located at the front and the back of the column, respectively. The foam height was monitored continuously for 1800 s by detecting the differences in light transmission through the glass column. For measuring the bubble size, a camera with a scanning area of 10.5 × 7.5 mm was positioned at a column height of 110 mm. The prism in the column allowed for obtaining images of the 2D foam structure. The resulting images were analyzed by the KRUSS Foam Analysis Software and the mean bubble area was recorded at every time step for 1800 s. The foams were characterized in triplicates.

#### 3.2.9. Surfactant Solution Cleaning by Filtration

After the foam cleaning process, the cleaning of the foaming surfactant solution was evaluated by the filtration technique. An amount of 50 mL of each surfactant solution was placed in sterile glass containers with lids. Each of the surfactant solutions was inoculated with 50 microliters of spore suspension at a concentration of 10^8^ CFU.mL^−1^ to mimic the contamination of the liquid phase of the foam after cleaning. Three samples of 5 mL from each surfactant solution were taken using a plastic syringe (Terumo, Leuven, Belgium) and they were filtered through a syringe filter (pore size: 0.2 µm, Sartorius, Goettingen, Germany). In order to control the spore concentration in the surfactant solutions, three samples of 100 μL each were taken from each of the surfactant solutions and serial dilutions were made in sterile Milli-Q water. Amounts of 100 μL of the filtered solutions and 100 μL of the serial dilutions were plated in Tryptic Soy Agar (TSA; Biokar Diagnostics, Allonne, France) and then incubated for 24 h at 30 °C. The number of bacterial colonies was counted, and the results were expressed as colony-forming units.mL^−1^ (CFU.mL^−1^).

#### 3.2.10. Life Cycle Assessment

The environmental impacts of the foam cleaning process using SDS and APGs were compared based on Life cycle assessment (LCA). The LCA methodology is standardized by ISO 14040 (2006) [27] and ISO 14044 (2006) [28]. The LCA consists of four phases:

- The objective and scope definition;
- The inventory analysis;
- The environmental impact assessment;
- The interpretation of the results.

The modeling of the LCA was performed using the Ecoinvent v3.0 database.

- The objective and scope definition

The objective of the LCA was to compare the environmental impacts of the foam cleaning process using SDS and the foam cleaning process using APGs. The functional unit (FU) is a unit of reference that allows for comparing different systems. In this study, the FU was defined as a cleaning of 0.05 m^2^ of the stainless-steel surface with at least 2 log CFU/cm^2^ of spores removal after 30 min of cleaning.

- The life cycle inventory

**Figure 9:**
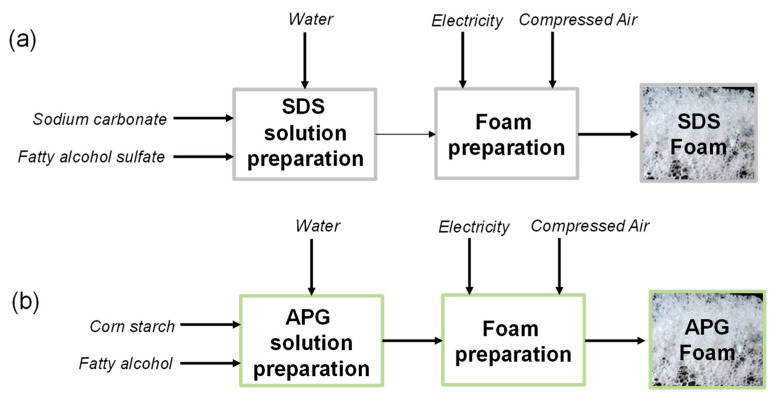
The process flowchart for the foam cleaning process formulated with: (**a**) SDS and (**b**) APG.

The process flowchart for foam cleaning with SDS and with APG is given in Figure 9. In both processes, the water consumption for foam production was 6 L.h^−1^ as the flow rate was 4.5 L.h^−1^, but an extra consumption was considered until the foam flow was stabilized. The consumption of compressed air to produce the foam was 6 L.h^−1^, and also an extra consumption was considered until the foam flow was stabilized. The electricity consumed by the pump to transport the surfactant solutions was 0.31 kW.h^−1^. The electricity consumption to compress the air was already taken into account in the compressed air data supplied by the Ecoinvent v3.0 database. Given the lack of data (in Ecoinvent) concerning the SDS and the APG, these substances were modeled (created) by using 40% sodium carbonate and 60% fatty alcohol sulfate for the SDS [11,25], and 59.7% potato starch and 40.3% fatty alcohol for the APG [29]. This study did not take into account transportation and electricity consumption for surfactant production.

- The impact assessment

In this study, the ReCiPe (v1.09) method was chosen for the impact assessment as it includes categories of relevance that are not included in other methods [30]. We chose the hierarchical perspective (H), as it considers the impacts in a more balanced way with respect to the temporal framework [31]. In order to focus on relevant data, a selection of environmental impacts was performed, based on their importance in other LCA studies that involve CIP in the food industry [11,30]. Eight environmental impacts were selected: climate change (kg.CO_2_ eq), marine eutrophication (kg.Neq), human toxicity (kg.1,4-DB eq), terrestrial ecotoxicity (kg 1,4-DB), marine ecotoxicity (kg.1,4-DB eq), natural land transformation (m^2^), water depletion (m^3^), and fossil resource depletion (kg.oil eq).

#### 3.2.11. Statistical Analysis

All the experiments were carried out in triplicate and the results were expressed as the mean ± standard deviation. The results were compared by one-way analysis of variance and Tukey’s test to analyze statistical differences (*p* < 0.05). The analysis was performed using SAS V8.0 software (SAS Institute, Gary, NC, USA).

## 4. Conclusions

This study showed that foams based on SDS and APGs could remove the spores deposited on model stainless-steel surfaces due to the foam wall friction mechanism. No effect on the APGs alkyl chain length between decyl glucoside and lauryl glucoside was observed. The foam properties were similar given the same effect on the foam flow removal of spores. After the foam cleaning process, the foams could be destabilized by ultrasound, and the spores be removed from the surfactant solutions by filtration, leading to a regeneration of the foam based on the same surfactant solutions. The LCA has shown that foam cleaning had significant environmental impact benefits with a reduction in water and energy consumption. The use of APGs helped to further decrease the environmental impacts in comparison to SDS in terms of natural land transformation, terrestrial ecotoxicity, marine ecotoxicity, human toxicity, fossil depletion, and climate change. Foams based on APGs were also more stable during the foam flow in comparison to foams based on SDS, which is an advantage to clean long ducts in the industry. In the future, it would be interesting to improve the foam stability by adding co-surfactants or polymers as already studied for foam flows in nuclear decontamination to try to improve further the spores’ removal action of the foam by increasing the foam friction mechanism and higher foam viscous stress [32]. Moreover, foams are already used to decontaminate static vertical surfaces from spores by using disinfecting formulations [16]. The next step in this study would be in the same way to combine the mechanical action of the foam flows with a chemical action to clean and decontaminate in the same time.

## Data Availability

The raw data will be available from the corresponding author upon reasonable request.

## Supplementary Materials

The following supporting information can be downloaded at: https://www.mdpi.com/article/10.3390/molecules28030936/s1, Table S1: Contribution of the foam cleaning process with APG and the foam cleaning process with SDS to the environmental impact categories selected according to the ReCiPe midpoint (H) impact assessment method.
