# Supplementary material for: Decontamination of Spores on Model Stainless-Steel Surface by Using Foams Based on Alkyl Polyglucosides
Source: Molecules. 2023 Jan 17;28(3):936. doi: 10.3390/molecules28030936 (PMC9919089; doi:10.3390/molecules28030936)
Supplement: Supplementary file 1 [file molecules-28-00936-s001.zip › molecules-2143761-supplementary.pdf]

Supporting Information

Decontamination of spores on model stainless steel surface by using foams based on alkyl polyglucosides

Carolina Dari [1], Heni Dallagi [1], Christine Faille [1], Thomas Dubois [1], Christelle Lemy [1], Maureen Deleplace [1], Marwan Abdallah [1], Cosmin Gruescu [1], Julie Beaucé [1], Thierry Benezech [1] and Anne-Laure Fameau [1,*]

[1] Univ. Lille, CNRS, INRAE, Centrale Lille, UMET, F-59000 Lille, France;

* Correspondence: anne-laure.fameau@inrae.fr

Table S1: Contribution of the foam cleaning process with APG and the foam cleaning process with SDS to the environmental impact categories selected according to the ReCiPe midpoint (H) impact assessment method.

Impact category	Unit	Foam Cleaning process with APG	Foam Cleaning process with SDS
Natural land transformation	m^2	1,30E-03	1,53E-03
Terrestrial ecotoxicity	Kg.1,4-DB eq	1,46E-03	1,62E-03
Climate change	kg CO_2 eq	2,58E+00	2,72E+14
Marine ecotoxicity	kg 1,4-DB eq	1,31E-02	1,48E-02
Human toxicity	kg 1,4-DB eq	5,59E-01	6,23E-01
Fossil depletion	kg oil eq	8,59E-01	9,31E-01
Water depletion	m^3	2,24E+01	2,27E+01
Marine eutrophication	kg N eq	7,25E-04	6,79E-04
